# Genetically modified mesenchymal stem cell therapy for acute respiratory distress syndrome

**DOI:** 10.1186/s13287-019-1518-0

**Published:** 2019-12-16

**Authors:** Jibin Han, Yuxiang Liu, Hong Liu, Yuanyuan Li

**Affiliations:** 1 Department of Critical Care Medicine, First Hospital of Shanxi Medical University, No. 85, Jiefangnan Road, Taiyuan, 030001 Shanxi China; 2 Shanxi Medical University, No.56, Xinjiannan Road, Taiyuan, 030001 Shanxi China

**Keywords:** Acute lung injury (ALI), Acute respiratory distress syndrome (ARDS), Mesenchymal stem cells (MSCs), Gene therapy, Genetic modification

## Abstract

Acute respiratory distress syndrome (ARDS) is a devastating hypoxemic respiratory failure, characterized by disruption of the alveolar-capillary membrane barrier. Current management for ARDS remains supportive, including lung-protective ventilation and a conservative fluid strategy. Mesenchymal stem cells (MSCs) have emerged as a potentially attractive candidate for the management of ARDS through facilitating lung tissue regeneration and repair by releasing paracrine soluble factors. Over the last decade, a variety of strategies have emerged to optimize MSC-based therapy. Among these, the strategy using genetically modified MSCs has received increased attention recently due to its distinct advantage, in conferring incremental migratory capacity and, enhancing the anti-inflammatory, immunomodulatory, angiogenic, and antifibrotic effects of these cells in numerous preclinical ARDS models, which may in turn provide additional benefits in the management of ARDS. Here, we provide an overview of recent studies testing the efficacy of genetically modified MSCs using preclinical models of ARDS.

## Background

Acute respiratory distress syndrome (ARDS) is a rapid onset of diffuse lung injury arising from a variety of insults, along with refractory hypoxemia ranging from mild to severe [1]. Currently, the Berlin criteria are considered a gold-standard for clinically defining ARDS [2]. Although findings gleaned from numerous studies in the past decade have provided more information about the biological basis of ARDS, there is a long way to reach a holistic understanding of the pathogenesis of ARDS. In terms of management, lung-protective ventilation, prone positioning, and conservative fluid strategies have been attempted to halt or reverse the course of ARDS [3–5]. Pharmacological interventions mainly target the inflammatory response to reverse the course of ARDS. Despite the encouraging results of preclinical studies, to date, no pharmacological-based therapies have been successfully translated to widely applicable interventions to manage ARDS in clinical settings [6–9]. Consequently, there is an urgent demand for more innovative and effective approaches to the management of ARDS. Mesenchymal stem cell (MSC) therapy is being considered as a promising intervention for treating ARDS due to the ability of MSCs to secrete a pool of trophic factors associated with anti-inflammation, angiogenesis, anti-fibrosis, and anti-apoptosis [10, 11]. Several favorable changes in the physiological and pathological features of acute lung injury (ALI) were observed in MSC-treated mice, including reduced lipopolysaccharide (LPS)-induced pulmonary inflammation and lung permeability compared to untreated mice [12, 13]. In addition, the human lung ex vivo model has also been tested, showing the therapeutic effects of MSCs in ALI.

## The general characteristics and identification of MSCs

MSCs, first termed osteogenic stem cells, were initially obtained from bone marrow by Friedenstein and colleagues in the 1960s and were shown to exhibit plastic-adherent, nonphagocytic, and fibroblastic characteristics [14]. In addition to the bone marrow, MSCs can also be isolated from various other sources, such as umbilical cord blood, amniotic fluid, placenta, dental pulp, liver, lung, thymus, synovium, and adipose tissues, offering various choices for their application [15]. Another feature of MSCs is their paracrine property of secreting various trophic factors and extracellular vesicles. Notably, there is overwhelming evidence that MSCs are a type of heterogeneous cell [16]. To solve this inconsistency with respect to the terminology and biologic properties, the International Society for Cellular Therapy (ISCT) suggests that researchers use mesenchymal stromal cells (MSCs) as the uniform nomenclature in all reports and proposes a minimal set of standard criteria for defining MSCs: primarily, MSCs have to adherence to plastic when in vitro cultured under standard condition; next, MSCs have to express CD105, CD73, and CD90 surface markers, and absence of CD45, CD34, CD14, CD11b, CD79, and CD19 on the cell surface; finally, MSCs have the capacity to differentiate into osteoblasts, adipocytes, and chondrocytes in the induction culture media [17, 18]. The ISCT proposed guidelines have been updated and now include changes in cell markers in response to activation [19].

## The mechanisms of action of MSCs in ARDS

**Fig. 1:**
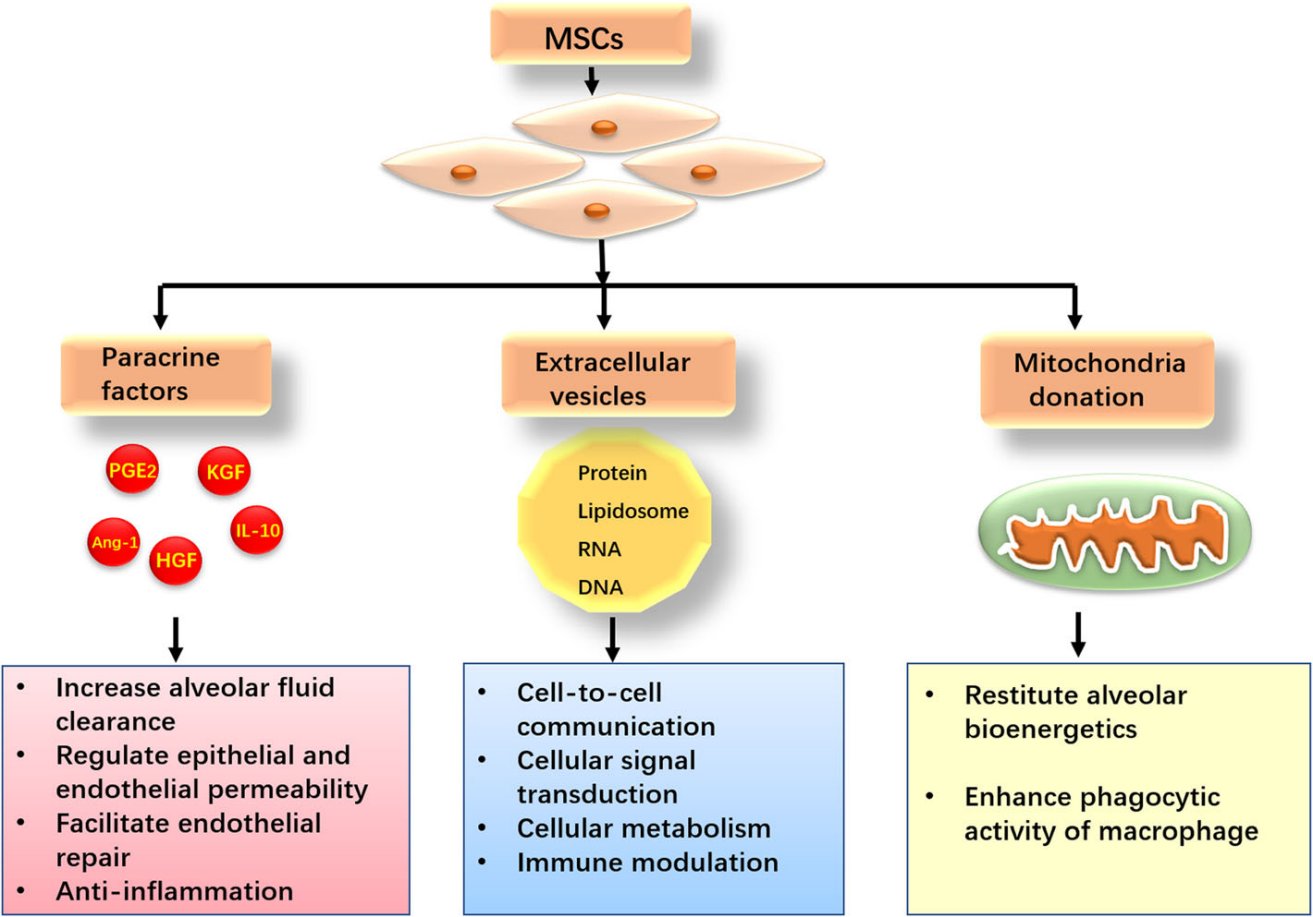
The mechanisms by which mesenchymal stem cells (MSCs) improve lung injury. MSCs secrete an abundance of paracrine soluble factors increase as well as releasing a great quantity of extracellular vesicles (EVs), exerting beneficial effects against lung injury. MSCs can also transfer mitochondria to the injured alveolar epithelium and macrophage, thereby restituting alveolar bioenergetics and enhancing phagocytic activity, respectively. KGF, keratinocyte growth factor; Ang-1, angiopoietin-1; PGE2, prostaglandin E2; IL-10, interleukin-10; HGF, hepatocyte growth factor; RNA, ribonucleic acid; DNA, deoxyribonucleic acid

Emerging studies support that the mechanism of action of MSCs responsible for injured tissue repair mainly depends on their paracrine property rather than differentiation [20]. MSCs secrete a plethora of paracrine soluble factors including keratinocyte growth factor (KGF), angiopoietin-1 (Ang-1), prostaglandin E2 (PGE2), interleukin-10 (IL-10), and other trophic cytokines. These paracrine factors can increase alveolar fluid clearance, regulate lung epithelial and endothelial permeability, facilitate endothelial repair, and reduce inflammation [21]. MSCs can also release a great quantity of extracellular vesicles (EVs), which envelop cytokines, growth factors, signaling lipids, functional mRNAs and microRNAs [22]. EVs participate in cell-to-cell communication, cellular signal transduction, cellular metabolism, and immune modulation both locally and at a distance in the body [23]. Furthermore, Islam et al. demonstrated that MSCs transferred mitochondria to the injured alveolar epithelium gave rise to increased alveolar ATP concentrations, thereby restituting alveolar bioenergetics and improving organ function [24]. In the in vitro and in vivo models of ARDS, the phagocytic activities of macrophages were enhanced after acquiring mitochondria from MSCs through tunneling nanotubes (TNT)-like structures, indicating that mitochondrial donation accounts for the bacterial clearance effect of MSCs in the setting of ARDS [25]. The mechanisms by which MSCs improve lung injury are illustrated in Fig. 1.

## Application of MSCs in patients with ARDS

Preclinical ARDS models using mice, rats, and sheep all support the safety and efficacy of MSC transplantation for the treatment of lung injury [26–28]. Of note, Lee et al. demonstrated the efficacy of MSCs in an ex vivo perfused human lung ALI model injured by *E. coli* endotoxin, as evidenced by restored alveolar fluid clearance and reduced extravascular lung water [29]. To date, MSC therapy in ARDS patients remains in its infancy, and there are two published phase I clinical trials that evaluated the safety of MSCs in patients with ARDS [30, 31]. Zheng et al. revealed that no patient suffered serious adverse events related to allogeneic adipose-derived MSC infusion during the 28-day study period [30]. Furthermore, Wilson et al. conducted a multi-center, open-label, dose-escalation phase I clinical trial of allogeneic bone marrow-derived human MSC administration for the treatment of patients with moderate-to-severe ARDS, and none of the MSC-related serious adverse effects were observed in this trial [31]. Recently, a prospective, double-blind, multi-center, randomized trial involving 60 participants also demonstrated that intravenous administration of MSCs was safe in patients with moderate to severe ARDS [32]. However, this study demonstrated that the 28-day mortality of the MSC group was numerically but not statistically higher than that of the placebo group, this tendency toward harm may correlated with MSCs viability [32]. It is worth noting that there are some limitations to these clinical trials. Firstly, the findings of these clinical studies are unreliable to assess efficacy due to the small sample size. Thus, it is very hard to distinguish whether the primary and secondary outcomes are correlated with MSC therapy or underlying critical illness. Moreover, all these clinical studies lacked data with respect to the time-response relationship of MSC therapy. It is still unclear about the frequency of MSCs administration should be performed in ARDS. Finally, there were no results related to the effect of MSC therapy on co-existing morbidities of the patients with ARDS. Registered clinical studies regarding the application of MSCs in patients with ARDS are summarized in Table 1.

**Table 1:** Registered clinical studies regarding the application of MSCs in patients with ARDS

ClinicalTrials.gov identifier	Study title	Study phase	Actual/estimated enrollment	Cell source	Cell does	Primary outcome	Location	Status
NCT01775774	Human mesenchymal stem cells for acute respiratory distress syndrome (START)	Phase 1	9 participants	Bone marrow MSCs	1 × 10^6^,5 × 10^6^, and 10 × 10^6^ cells/kg	Infusion associated adverse events	USA	Completed
NCT03608592	Human umbilical cord-derived mesenchymal stem cells therapy in acute respiratory distress syndrome	Phase 1	12 participants	Umbilical cord-derived MSCs	1 × 10^6^ cells	Infusion associated events	China	Not yet recruiting
NCT02804945	Mesenchymal stem cells (MSCs) for treatment of acute respiratory distress syndrome (ARDS) in patients with malignancies	Phase 1	20 participants	Bone marrow MSCs	3 × 10^6^/cells/kg	Adverse events	USA	Active, not recruiting
NCT01902082	Adipose-derived mesenchymal stem cells in acute respiratory distress syndrome	Phase 1	20 participants	Adipose-derived MSCs	1 × 10^6^/cells/kg	Adverse events	China	Completed
NCT02444455	Human umbilical-cord-derived mesenchymal stem cell therapy in acute lung injury (UCMSC-ALI)	Phase 1/2	20 participants	Human umbilical cord MSCs	5 × 10^5^/cells/kg	Major adverse events	China	Recruiting
NCT02112500	Mesenchymal stem cell in patients with acute severe respiratory failure (STELLAR)	Phase 2	10 participants	Bone marrow MSCs	Unclear	Oxygen index at 3 days after MSCs infusion	Korea	Unknown
NCT03042143	Repair of acute respiratory distress syndrome by stromal cell administration (REALIST)	Phase 1/2	75 participants	Umbilical cord-derived MSCs	Unclear	Oxygenation index, serious adverse events	UK	Recruiting

## Genetic modification strategy to improve therapeutic potential of MSCs

Due to the lack of randomized phase III/IV clinical trials, we cannot draw any definitive conclusions concerning the therapeutic effects of MSCs in patients with ARDS at present. To provide incremental benefits in the management of ARDS, MSCs genetically engineered to produce desirable trophic cytokines or other beneficial gene products have been explored to further optimize the therapeutic efficacy of MSCs in numerous preclinical models [33–35]. Genetic modification of MSC is commonly carried out with viral or non-viral transfection methods.

### Viral transduction

Viral transduction is the most efficient way to integrate exogenous genes into MSCs, while not compromising the self-renewal and differentiation capacity of the progeny [36]. Experienced research personnel can commonly obtain 90% transfection efficiency of target gene in MSCs via viral transfection [37]. Lentiviral vector, adenoviral vector, and adeno-associated virus (AVV) are the most common viral vectors. Lentiviral transfection can enable stable transgene expression levels in the target cells. Adenoviral vector affords merely transient expression of targeting genes on MSCs. AAV is the absence of immunogenic in murine models and generates sustained transgene expression. However, there are some concerns related to viral transfection technique. First and foremost, viral transfection carries a risk of activation of oncogenes and results in tumorigenesis. Furthermore, viral vectors may trigger adverse immune reactions and weaken the stability of transgene after administration [38]. For instance, a great proportion of the population with pre-existing neutralizing antibodies against AAV, which result in a decline in gene transfer efficacy in vivo [39].

### Non-viral transfection

Non-viral transfection techniques are divided into physical methods and chemical methods [40]. Although non-viral transfection methods have many advantages, including easy to scale up, low production cost, low risk of adverse immune reactions, and are versatile with a variety of experimental protocols, there are drawbacks to these techniques [41]. Physical methods contain electroporation and ultrasound sonoporation. Electroporation can transfer the nucleic acid into the cytoplasm through the cellular pores opened by electrical pulse, but this handling procedure results in considerable cell death. Otani et al. have shown that the combination of ultrasound and microbubbles (US-MB) can deliver small interfering RNA (siRNA) into MSCs, whereas the high-intensity supersonic markedly decreased the viability of MSCs [42]. Liposomes are the most commonly used chemical methods, but the transfection efficiency is low, typically transfecting merely 2–35% of MSCs [43].

## Application of gene-modified MSCs in preclinical ARDS models

As mentioned above, the genetically modified MSC approach is particularly valued due to its ability to target genes of interest within injured tissues, displaying promise for clinical translation.

### Genetically modified MSCs to improve homing

MSCs have the ability to home to the site of damage so systemic delivery of MSCs is the most prevalent route and is widely used in clinical practice [44]. However, MSCs express a relatively low levels of homing molecules and extensive expansion means the homing receptors can be lost, thus the in vivo homing of MSCs to the target tissue is not sufficient, which in turn hampers their therapeutic efficacy [45]. Accordingly, genetically modified MSCs with candidate gene is considered a promising way to improve their homing ability. A study demonstrated that MSCs preferably migrated to the damaged tissue after administration [46], thereby contributing to the restoration of pulmonary structure and function. The efficacy of MSC therapy is critically dependent on efficient cell delivery [47]. Extensive expansion is required to harvest a great quantity of MSCs for systemic administration. However, culture-expanded MSC loose an abundance of homing receptor. Therefore, the low homing rate of MSCs is a common occurrence after systemic infusion; this phenomenon has been observed in many disease models [48, 49]. The most crucial issue is to ensure that adequate numbers of transplanted cells reach the target tissue by enhancing their migratory ability while minimizing the risk of embolism. MSCs migrate to sites of injury through the mechanism of mimicking leukocyte recruitment in an inflammatory setting, and chemokines are believed to play a pivotal role in promoting the homing of injected MSCs to target tissues [49]. In particular, stromal cell-derived factor-1 (SDF-1), a ligand for the chemokine receptor (CXCR4), is a critical chemokine of cellular migration, attracting CXCR4-expressing cells to the site of tissue injury [50]. Moreover, there was an increase in the level of SDF-1a following lung injury. However, the surface levels of the receptor CXCR4 gradually decreased as MSCs expanded, ultimately limiting the efficacy of MSCs [51, 52]. Therefore, it may be expected that CXCR4 genetically modified MSCs can improve the homing efficiency. To test this hypothesis, Yang et al. overexpressed CXCR4/GFP or GFP in MSCs using a lentiviral vector and subsequently investigated them in a rat model of ALI injured by LPS, demonstrating that CXCR4-MSCs showed improved homing and colonization of injured lung tissue compared with the control GFP-MSCs, which resulted in a decrease in the levels of pro-inflammatory cytokines and neutrophil numbers in bronchoalveolar lavage fluid (BALF) [53]. A similar study manipulated the homing property of MSCs through lentiviral-mediated modification of the E-prostanoid 2 (EP2) receptor, and the immunofluorescence staining showed that more EP2-MSCs were observed in the site of lung injury than control MSCs [54]. However, these preliminary results were obtained from mouse models of lung injury induced by LPS, which cannot fully recapture the pathophysiological characteristics of patients with ARDS. Thus, the implementation of genetic modification to facilitate MSC homing to the site of the damaged lung tissue should be validated in ARDS models induced by a variety of causative origins before translation into clinical ARDS settings.

### Genetically modified MSCs to facilitate anti-inflammation

To date, two distinct sub-phenotypes of ARDS have been reported, including hyperinflammatory sub-phenotype and hypoinflammatory sub-phenotype, respectively [55, 56]. ARDS patients with hyperinflammatory sub-phenotype are featured by exuberant inflammation [56]. Another attractive feature of MSCs relates to their anti-inflammatory properties [57]. Genetic engineering is also used to further enhance the inherent anti-inflammatory action of MSCs. IL-1 receptor-like-1 (ST2) is known to be a decoy receptor for interleukin-33 (IL-33), which is released after epithelial or endothelial injury [58]. Growing evidence suggests that the IL-33/ST2 axis plays a role in tissue damage and innate airway inflammation via initiating and enhancing both the Th1 and Th2 immune responses [59]. Human MSCs overexpressing sST2 via lentiviral transfection have demonstrated incremental therapeutic benefits in a murine model of ALI [60]. In this study, overexpression of sST2 provided additional anti-inflammatory effects, evidenced by a considerable reduction in the numbers of inflammatory cells and neutrophils in the lung airspace, along with a significant decrease in the concentrations of pro-inflammatory cytokines (TNF-α and IL-6) in the BALF [60]. Angiotensin II (Ang II) is known to participate in the development of ARDS via initiating the lung inflammatory response [61]. Angiotensin-converting enzyme 2 (ACE2) can facilitate the degradation of AngII into Ang1–7, thereby alleviating the pro-inflammatory effect of AngII in ARDS [62]. Thus, the anti-inflammatory property of MSCs was also manipulated via overexpression of the ACE2 gene. He et al. used lentiviral transduction of ACE2 in MSCs followed by intravenous delivery of MSCs-ACE2 in a mouse model of ALI induced by LPS [63]. Their findings suggested that mice treated with MSCs-ACE2 demonstrated a significant reduction in neutrophil influx and pro-inflammatory cytokine levels when compared to mice treated with unmodified MSCs. These incremental anti-inflammatory effects were deemed related to the accelerated degradation of Ang II as a consequence of the increased level of ACE2 in injured lung tissues. However, it is noteworthy that inflammation is not always detrimental; inflammation also plays a vital role in the repair process and resolution of ARDS. Accordingly, anti-inflammatory properties enhanced by genetically modified MSCs might be deleterious in the late stage of ARDS, leading to immunosuppression or immunoparalysis. Additionally, genetically modified MSCs to facilitate anti-inflammation may be harmful for patients with ARDS hypoinflammatory sub-phenotype.

### Genetically modified MSCs to repair the alveolar-capillary barrier

Disruption of the alveolar-capillary barrier is partly related to non-cardiogenic pulmonary edema following ARDS [64]. Alveolar epithelial fluid transport is critical for the recovery of pulmonary function [65]. For this reason, accelerating alveolar epithelial recovery has been reviewed as a target in the treatment of ARDS. Keratinocyte growth factor (KGF) is one of the most powerful cytokines that participates in the process of pulmonary epithelial healing by promoting alveolar type II cell proliferation and stimulating surfactant synthesis, thereby contributing to edema clearance [66]. In a murine model of LPS-induced ALI, the intravenous application of MSCs overexpressing KGF led to a significant decrease in pulmonary edema compared to the vehicle control, as quantified by measuring the total protein in bronchoalveolar lavage fluid [67].

In addition, ARDS is characterized by bilateral pulmonary edema resulting from disruption of the endothelial barrier [68], highlighting the need for novel therapeutic strategies targeting the pulmonary endothelium. MSCs display substantial pro-angiogenic properties mainly via the secretion of angiogenic factors, such as vascular endothelial growth factor (VEGF), hepatocyte growth factor (HGF), and insulin-like growth factor 1 (IGF-1), and these cytokines can act individually or synergistically to promote the recovery of the pulmonary vasculature [69]. Although it is widely accepted that MSCs participate in angiogenic processes during tissue repair and regeneration by producing multiple soluble factors, including VEGF and HGF, the level of these angiogenic factors secreted by MSCs in vitro is too low to be detected and thus cannot rapidly promote the angiogenic response after lung injury [70]. HGF was originally considered a potent mitogen for hepatocytes; however, emerging evidence suggests that HGF also actively participates in angiogenesis [71]. Adenoviral vectors were used to make MSCs overexpress HGF offered an incremental potential in promoting revascularization following radiation-induced lung injury [72]. A recent study suggested that VEGF produced by MSCs synergized with HGF in stabilizing endothelial cell barrier function in an in vitro co-culture model [73]. Furthermore, VEGF gene knockdown in MSCs reduced the protective effect of MSCs on lung vascular permeability in a rat model of LPS-induced ALI, indicating that VEGF is required for MSCs to exert the angiogenesis effect [74]. In addition, the Angiopoietin-1 (Ang-1) is also considered a target to enhance the pro-angiogenic properties of MSCs. McCarter et al. assessed the therapeutic potential of Ang-1 overexpression in a rat model of ALI injured by LPS [75]. Their results showed that fibroblast cells transfected with plasmid vector containing human Ang-1 resulted in marked improvement in endothelial integrity. In addition, they also reported that the Ang-1/Tie2 axis plays a crucial role in maintaining pulmonary vascular stabilization following lung injury, as demonstrated in Ang-1-overexpressing or Tie2-deficient transgenic mice. As a ligand of endothelial Tie2, Ang-1 is a strong pro-angiogenic mediator with the capacity to protect vascular endothelium against plasma leakage [76–78]. Karmpaliotis et al. demonstrated that downregulation of Ang-1 was associated with decreased capillary leakage and neutrophil infiltration [79]. Considering the anti-permeability and endothelial-protective features of the Ang-1 gene, MSCs were engineered to overexpress Ang-1 using a lentivirus vector and were used to treat LPS-induced lung injury, demonstrating a further improvement in pulmonary vascular endothelial permeability [80]. Moreover, Mei et al. demonstrated that MSCs transfected with the Ang-1 gene were significantly more effective than MSCs alone in a murine model of LPS-induced ALI, providing additive benefits in both pulmonary vascular permeability and alveolar inflammation, as evaluated by reductions in albumin and pro-inflammatory cytokine levels in BAL, respectively [81]. Taken together, these findings imply that genetic modification of MSCs may be beneficial to further attenuate permeability of the alveolar-capillary barrier and subsequently improve arterial oxygenation for critically ill patients with ARDS. Nevertheless, the therapeutic potential of MSCs depends largely on passage, as extended handing of MSCs and genetic modification in vitro may significantly diminish their ability to protect the pulmonary alveolar-capillary barrier due to cellular senescence.

### Genetically modified MSCs enhance anti-apoptosis

Abundant reactive oxygen species are generated during the pathophysiological process of ARDS, subsequently facilitating the process of cell apoptosis, ultimately leading to deterioration in pulmonary function [82]. Accordingly, another transgenic expression strategy to augment the efficacy of MSCs is to overexpress genes with protective effects against apoptosis. Manganese superoxide dismutase (MnSOD) is the chief reactive oxygen species (ROS) scavenging enzyme that counteracts ROS overproduction, by degrading superoxide radicals into oxygen and hydron peroxide, consequently protecting cells from the deleterious action of ROS overproduction [83]. It is noteworthy that MSCs overexpressing MnSOD could protect against apoptosis induced by tert-butyl hydroperoxide [84]. In mouse models of radiation-induced lung injury (RILI), MSCs overexpressing MnSOD significantly facilitate the improved recovery of RILI, mainly attributable to MnSOD-MSCs protecting the lung cell from apoptosis, as measured by terminal deoxynucleotidyl transferase-mediated nick-end labeling (TUNEL) immunohistochemical staining [85]. Heme oxygenase-1 (HO-1) is a cytoprotective enzyme with anti-apoptotic property. The study performed by Chen et al. demonstrated that HO-1-modified bone-marrow-derived MSCs have an additional protective effect against lung injury than MSCs delivery alone [86]. It is worth noting that a dysfunction in neutrophil apoptosis in the inflammatory phase of ARDS paradoxically can damage host tissues [87]; therefore, the strategy with genetically modified MSCs to enhance anti-apoptosis may be detrimental. Research regarding the application of gene-modified MSCs in preclinical ARDS models are summarized in Table 2.

**Table 2:** Research regarding application of gene modified MSCs in preclinical ARDS models

Candidate gene	Engineering vector	Animal species	ARDS model	MSC source	MSC species	MSC dose	Administration route	References
CXCR4	Lentiviral vector	Rat	LPS-induced	Bone marrow	Rat	1 × 10^6^	Tail vein	[53]
EP2	Lentiviral vector	Mice	LPS-induced	Bone marrow	Mice	5 × 10^5^	Tail vein	[54]
sST2	Lentiviral vector	Mice	LPS-induced	Adipose tissue	Human	1 × 10^6^	Tail vein	[60]
ACE2	Lentiviral vector	Mice	LPS-induced	Bone marrow	Mice	5 × 10^5^	Tail vein	[63]
KGF	Lentiviral vector	Mice	LPS-induced	Bone marrow	Mice	5 × 10^5^	Tail vein	[67]
HGF	Adenoviral vector	Mice	radiation-induced	Bone marrow	Human	1 × 10^6^	Tail vein	[72]
Ang-1	Lentiviral vector	Mice	LPS-induced	Bone marrow	Mice	1 × 10^5^	Jugular vein	[79]
Ang-1	Nuclear-targeting electroporation	Mice	LPS-induced	Bone marrow	Mice	2.5 × 10^5^	Jugular vein	[80]
MnSOD	Lentiviral vector	Mice	radiation-induced	Bone marrow	Human	1 × 10^6^	Tail vein	[85]
HO-1	Lentiviral vector	Rat	LPS-induced	Bone marrow	Rat	5 × 10^5^	Tail vein	[86]

*ARDS* acute respiratory distress syndrome, *CXCR4* chemokine receptor 4, *LPS* lipopolysaccharide, *EP2* E-prostanoid 2, *sST2* soluble IL-1 receptor-like-1, *ACE2* angiotensin-converting enzyme 2, *KGF* keratinocyte growth factor; HGF, hepatocyte growth factor, *Ang-1* angiopoietin-1, *HO-1* heme oxygenase-1, *MnSOD* manganese superoxide dismutase

## Concerns regarding MSC-based gene therapy in ARDS

Although the genetically modified MSC strategy has shown promising results in preclinical animal models of ARDS, the application of genetically modified MSCs for translational purposes appears much more hypothetical and uncertain; there are still some concerns that need to be addressed in the near future. First, there is a concern that genetic manipulation may have an impact on the functional characteristics of MSCs, including their tri-lineage differentiation capability, immunosuppressive properties, and cell surface receptor phenotype. Second, the therapeutic use of genetically modified MSC therapy exposes the genetic instability that can be induced by the genetic manipulation of MSCs, and little information is available regarding the long-term behavior of genetically modified cells in vivo after systematic or local administration. Additionally, it is important to note that diverse vectors have been applied to transfect MSCs, a variety of MSC sources have been used, various models of preclinical ARDS have been studied, and different methods have been used to evaluate the therapeutic efficacy in these studies, so caution should be used when interpreting these findings. Moreover, from the clinical application perspective, it may be difficult to rapidly generate enough genetically modified MSCs within a few days to transplant following the onset of ARDS. In the vast majority of the published studies, researchers investigated only the overexpression of a pre-selected gene of interest to improve MSC therapy; however, it is justifiable to suppose that the expression of certain genes can blunt the therapeutic effects of MSCs; therefore, it might be interesting to knockdown/knock out genes in MSCs to enhance the therapeutic potency of MSCs in ARDS.

## Conclusions

In conclusion, MSCs are being exploited as a gene delivery vehicle to manage ARDS due to their low immunogenicity, their pleiotropic effects, and their preferential migration to the site of damage. Although a genetic engineering strategy aimed at improving the therapeutic efficacy of MSCs by either increasing cell delivery or the effective dose of trophic factors to the damaged host tissue is showing promise in preclinical studies, challenges remain. Lacking specific surface markers to assess the quality of MSCs obtained from different origins, paracrine factor profiles of MSCs vary with their tissue origin, and functional heterogeneity of MSCs exists between individuals are obstacles faced by clinical translation. In addition, no standardized protocol is established for genetic modification of MSCs may result in a high degree of variability in their proliferation and differentiation potential. Accordingly, genetic modification of MSC may accompany safety concern and result in unpredictable clinical consequences. Particularly, patients with ARDS in different stages or sub-phenotypes may have different treatment responses to genetically modified MSC therapy. Future studies are needed to overcome these scientific hurdles before genetically modified MSC therapy can be translated into clinical practices for patients with ARDS.

## Data Availability

Not applicable.

## Abbreviations

ACE2: Angiotensin-converting enzyme 2
ALI: Acute lung injury
Ang II: Angiotensin II
Ang-1: Angiopoietin-1
ARDS: Acute respiratory distress syndrome
BALF: Bronchoalveolar lavage fluid
CXCR4: Chemokine receptor 4
EP2: E-prostanoid 2
HGF: Hepatocyte growth factor
HO-1: Heme oxygenase-1
IGF: Insulin-like growth factor 1
IL-10: Interleukin-10
KGF: Keratinocyte growth factor
MnSOD: Manganese superoxide dismutase
MSCs: Mesenchymal stem cells
PBW: Predicted body weight
PGE2: Prostaglandin E2
ROS: Reactive oxygen species
SDF-1: Stromal cell-derived factor-1
sST2: Soluble IL-1 receptor-like-1
TUNEL: Terminal deoxynucleotidyl transferase-mediated nick-end labeling
VEGF: Vascular endothelial growth factor
